# Dr. James Webster Knapp

**Published:** 1915-08

**Authors:** 


### OBITUARY

**Readers are requested to report promptly the death of all physicians in Western New York, or former residents of this region, or graduates of any medical school in Western New York, and to notify the families of the deceased of our desire to publish adequate obituary notices.**

**Dr. James Webster Knapp,** Syracuse 1881, died suddenly at his home in Canastota, N. Y., June 26, aged 62. He was surgeon to the N. Y. Central and Oneida R. R.'s, president of the Canastota Hospital and of the trustees of the Canastota Library, and was a member of the County, State and National societies.

